# Pharmacokinetics, safety, and tolerability of a depot formulation of naltrexone in alcoholics: an open-label trial

**DOI:** 10.1186/1471-244X-5-18

**Published:** 2005-04-01

**Authors:** Gantt P Galloway, Monika Koch, Ryan Cello, David E Smith

**Affiliations:** 1 Haight Ashbury Free Clinics, San Francisco, USA; 2 The Permanente Medical Group, Chemical Dependency and Recovery Program, Vallejo, USA; 3 Department of Clinical Pharmacy, University of California San Francisco, San Francisco, USA

## Abstract

**Background:**

Naltrexone is an effective medication for treatment of alcohol dependence, but its efficacy is limited by lack of adherence to the oral dosage form. A long-acting depot formulation of naltrexone may increase adherence.

**Methods:**

A single site, 6-week open label study was conducted with 16 alcohol dependent subjects each receiving 300 mg of Naltrexone Depot by intramuscular injection. The main outcomes were safety and tolerability of the Naltrexone Depot formulation, blood levels of naltrexone and its main metabolite 6-beta naltrexol, and self-reported alcohol use. All subjects received weekly individual counseling sessions.

**Results:**

The medication was well tolerated with 88% of subjects completing the 6-week trial. The most common side effect experienced was injection site complications. There were no serious adverse events. Subjects had naltrexone and 6-beta-naltrexol concentrations throughout the trial with mean values ranging from 0.58 ng/mL to 2.04 ng/mL and 1.51 ng/mL to 5.52 ng/mL, respectively, at each sampling time following administration. Compared to baseline, subjects had significantly reduced number of drinks per day, heavy drinking days and proportion of drinking days.

**Conclusion:**

Naltrexone Depot is safe and well tolerated in alcoholics and these findings support the further investigation of its utility in larger double-blind placebo controlled trials.

## Background

Naltrexone is an opiate receptor antagonist that was approved in 1984 for the treatment of opiate dependence. The safety and efficacy of naltrexone in reducing alcohol consumption were established in controlled clinical trials [1,2] which led, in 1994, to United States Food and Drug Administration (FDA) approval of a 50 mg oral tablet for treatment of alcohol dependence. Since receiving FDA approval, several studies on naltrexone's effectiveness in reducing alcohol consumption have been conducted. Naltrexone has demonstrated effects on drinking behavior in alcoholics through reducing alcohol use among subjects who sample alcohol as well as promotion of alcohol abstinence [3-5]. One study that used cognitive behavior therapy and random assignment to oral naltrexone or placebo in recently abstinent alcoholics found that 62% of the naltrexone group did not relapse into heavy drinking in comparison with 40% of the placebo treated subjects [4]. In the laboratory setting, naltrexone has been shown to increase the latency to drink alcohol in social drinkers [6], to reduce drinking in heavy drinkers [7] and increase certain discriminant and sedative effects of ethanol while reducing the positive reinforcing effects of ethanol in non-alcoholics [8].

While many single site studies and 2 meta-analyses [9,10] indicate that naltrexone is more effective than placebo, there have also been 2 multisite studies and 1 single site study in which naltrexone was not found to be effective in decreasing alcohol consumption [11-13].

One factor that appears to be important for naltrexone's effectiveness is adherence. In 2 studies, oral naltrexone was found to have an effect only in the population of patients who were highly compliant with their medication [14,15]. Alcoholics have been shown to have particularly low rates of medication adherence [16,17]. With treatments intended to reduce or prevent relapse to alcohol or drug abuse, non-adherence has an additional aspect. If a patient on naltrexone wants to resume drinking or using drugs, they can discontinue their medication and experience the full effect of the drug.

In alcoholics, an injectable sustained release formulation (SRF) would be highly advantageous. A SRF of naltrexone would reduce the number of opportunities to impulsively discontinue their medication and ensure that discontinuation came to the attention of the health care provider scheduled to administer the injection. A SRF would minimize the number of doses required and guarantee exposure to the medication for at least the duration of the first injection. It would have the added benefit of producing a more consistent and predictable drug blood level since a depot injection bypasses first pass metabolism [18].

Kranzler et al. [5] conducted a 12-week study of a different depot formulation of naltrexone in 20 alcohol dependent subjects. Subjects received either a subcutaneous injection of 206 mg NTX (N = 15) or placebo (N = 5) along with eight weekly coping skills sessions. Compared to placebo, subjects who received the SRF of naltrexone had fewer heavy drinking days. These initial results support the continuation of research into the use of a SRF of naltrexone in patients being treated for alcohol dependence.

Comer et al. [19], evaluated the effectiveness, time course and safety of the same naltrexone depot formulation used in Kranzler's study [5], in 12 heroin dependent subjects over an 8 week time span. The results from this study showed that blood plasma levels remained above 1 ng/mL for 3–4 weeks after receiving either 192 mg or 384 mg of naltrexone depot. In this study there were few adverse events reported other than the discomfort associated with the actual injection of the depot formulation. It was shown that the depot formulation of naltrexone at both doses provided safe, effective and long lasting antagonism of the effects of heroin.

The current study, a single site open-label study, was designed to examine the safety, pharmacokinetics and tolerability of the DrugAbuse Sciences (DAS; Hayward, CA) Naltrexone Depot formulation for treatment of alcohol dependence. During the study, subjects received 1 depot injection of naltrexone and 6 weeks of individual counseling.

## Methods

### Study design

Sixteen subjects participated in this 6-week, single site, open-label study to investigate the safety and tolerability of Naltrexone Depot along with naltrexone and 6-beta-naltrexol blood levels. The study was conducted at Friends Research Associates in Berkeley, CA. All procedures were approved by the Institutional Review Board of Friends Research Institute.

**Figure 1:**
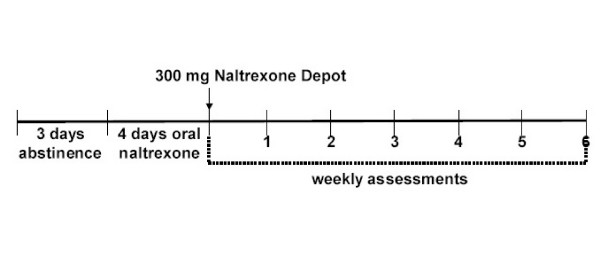
Study design of naltrexone depot in alcohol dependence.

Following initial screening, subjects were required to have 3 days of abstinence from alcohol. Subjects who tolerated four days of oral naltrexone returned to the clinic the day following their last dose of oral naltrexone and received intramuscular injections of Naltrexone Depot containing 300 mg of naltrexone. See figure 1 for an outline of study procedures.

### Subjects

Subjects were recruited through advertising. Subjects were males and non-pregnant, non-nursing females, age 18 to 65, with DSM-IV diagnoses of alcohol dependence who expressed a desire to stop drinking. To meet inclusion criteria, subjects had to have had at least 1 day of heavy drinking (≥ 5 drinks/day for males or ≥ 4 drinks/day for females) within the preceding 14 days. As noted above, they had to be able to achieve at least three consecutive days of sobriety, without detoxification medications. Female subjects were required to practice effective birth control for the duration of the trial and all subjects were required to provide a urine sample negative for amphetamines, barbiturates, benzodiazepines, cocaine and opiates.

Subjects were excluded from the study if they were currently taking disulfiram, naltrexone or a neuroleptic medication; if they needed medical detoxification from alcohol; or if they had a DSM IV diagnosis of dependence on any drug of abuse other than nicotine or alcohol. Subjects were also excluded if they had an ALT or AST elevation more than 3 times the upper limit of normal or any psychiatric condition (e.g., depression with suicidal ideation) or medical condition that would preclude safe participation in the protocol. A complete list of inclusion and exclusion criteria is presented in Table 1.

**Table 1:** Inclusion and Exclusion Criteria

**Inclusion Criteria**
• Males or non-pregnant, non-nursing females, age 18 to 65, with a DSM-IV diagnosis of alcohol dependence who express a desire to stop drinking.
• Heavy drinking (5 drinks/day for males or 4 drinks/day for females) within 14 days prior to randomization
• Able to achieve at least three continuous days of sobriety, without detoxification medications, immediately before beginning the oral naltrexone run-in dosing
• Willing and able to give informed consent
• Willing to practice effective birth control for duration of trial (female patients only)
• Available to participate in the study for 7 weeks
• Willing to provide names and permission to contact someone (e.g., spouse, parent, friend) who would likely know their whereabouts for follow-up tracking
• Naltrexone tolerance as demonstrated during run-in dosing period.

**Exclusion Criteria**

• Currently taking disulfiram (Antabuse), naltrexone (Revia or generic) or neuroleptic medication
• In need of medical detoxification from alcohol
• DSM-IV diagnosis of dependence on any drug of abuse other than nicotine or alcohol or drug screen showing benzodiazepines, marijuana, cocaine, methamphetamine, barbituates or heroin
• Clinical evidence of cardiac ischemia (by EKG or medical history) or history of myocardial infarction within the previous 2 years
• History of pancreatitis
• Planned surgery within 7 weeks of screening
• Any chronic or episodic painful conditions that would reasonably require opiate medications for pain control
• ALT or AST elevations more than 3 times the upper limit of normal
• Subjects with any psychiatric (e.g., depression with suicidal ideation) or medical condition that would preclude safe participation in the protocol.
• History of allergic or adverse response to naltrexone
• Participation in a trial of an investigational medication within 30 days prior to study enrollment
•Subjects mandated by court for alcohol or drug abuse treatment or having pending legal proceedings that could result in incarceration within 7 weeks of screening

### Study procedures

Before participation in study procedures began, prospective subjects had the study explained to them by a member of the research team. The purpose of the study was reviewed, and the potential risks and discomforts of study participation were explained. Subjects were given a copy of an informed consent form to read. Just prior to signing the consent form a Breathalyzer test was performed; subjects were not able to sign the consent unless their BAL was less than 0.04 mg/dL. Following signing of the consent form, a medical history and physical examination were conducted to assess eligibility for study participation. In addition, subjects had an EKG performed along with screening hematology, clinical chemistry, serum pregnancy test (for women of childbearing age), urine toxicological screen and a routine urinalysis.

Following screening, subjects were administered one 50 mg naltrexone tablet at the clinic. Three additional tablets were dispensed for the subjects to take on their own (1 each day for 3 days). Subjects then returned to the clinic site the day following the final naltrexone dose. Subjects who tolerated the oral naltrexone were eligible to participate in the study. The rationale for an oral naltrexone run-in was to avoid exposing naltrexone intolerant individuals to Naltrexone Depot, which once it is given cannot be removed. Eligible subjects received 2 intramuscular injections of 150 mg of Naltrexone Depot, one in each buttock, for a total dose of 300 mg.

Subjects then received brief counseling following their injection and at each weekly study visit throughout the 6-week trial. A therapist provided a manualized form of counseling called BRENDA. BRENDA is an acronym for a six step approach to counseling: **B**iopsychosocial evaluation, **R**eport to patient on assessment, **E**mpathetic understanding of the patients problems, **N**eeds expressed by the patient which should be addressed, **D**irect advice on how to meet those needs, and **A**ssess response/behaviors of patients to advise and adjust treatment recommendations. BRENDA was chosen because, with treatment of alcohol and drug abuse increasingly occurring in the context of mainstream medical care, it is important to use brief counseling therapies that are feasible within these settings [20,21]. Subjects were permitted but not required to attend Alcoholics Anonymous (AA) or other self-help recovery programs.

### Outcome measures

Each week, subjects were provided with a drinking diary. In the diary, subjects were asked to record all of the alcoholic drinks that they consumed and to return to clinic with the completed diary each week. At each weekly visit, the research assistant reviewed the diary with the subject and recorded adverse events, service utilization and drinking history. Timeline follow-back procedures were used [22-25]. Using timeline follow-back procedures, the number of standard drinks per day was recorded for each day since the last visit. If a subject missed a data collection visit, the timeline follow-back was extended to the last visit attended. Subjects rated their maximum craving for alcohol during the past week on a visual analog scale. The research assistant also queried subjects about alcohol craving and concurrent medications. Subjects had their injection sites examined, a Breathalyzer test performed, a blood draw taken and a urinalysis collected during each weekly visit. The blood was analyzed for naltrexone and 6-beta-naltrexol plasma levels along with GGT levels.

An LC-MS/MS method was used to determine plasma naltrexone and 6-beta-naltrexol levels simultaneously. A liquid/liquid extraction under basic conditions was done before injection into the LC-MS/MS. A PE Sciex API III+, using an electrospray interface, was employed in this study. Four analytical runs were required to process the samples from this study. Naltrexone and 6-beta-naltrexol analyses were performed by MDS Pharma Services, Lincoln, NE.

The linear range for naltrexone was 0.100 to 50.000 ng/mL with a limit of quantitation of 0.100 ng/mL. Naltrexone quality control samples analyzed with each analytical run had coefficients of variation less than or equal to 6.14% and absolute relative errors less than or equal to 7.67%. The linear range for 6-beta-naltrexol was 0.400 to 100.000 ng/mL with a limit of quantitation of 0.400 ng/mL. 6-beta-naltrexol quality control samples analyzed with each analytical run had coefficients of variation less than or equal to 7.40% and absolute relative errors of less than or equal to 7.70%.

Drinking measures were: drinking days (days on which any drinking occurred), heavy drinking days (≥ 5 drinks/day for men; ≥ 4 drinks/day for women) and drinks per drinking day. These measures were cumulated for the 14-day baseline period and for the 6 weeks following the naltrexone injection.

Alternate measures were generated for days on which drinking data were not obtained after naltrexone was administered. These missing days were counted as both drinking days and heavy drinking days and the number of drinks per drinking day were set equal to the mean number of drinks per drinking day during the baseline period. Analyses using these alternate measures did not yield significantly different results and are not presented.

### Study medication

Naltrexone is a competitive antagonist at the mu opiate receptor. Following oral administration naltrexone is almost completely absorbed, but subject to first pass metabolism; the bio-availability ranges from 5% to 60% [26,27]). Naltrexone is primarily eliminated by the liver with only 1% of an oral naltrexone dose excreted in urine [28]. Conjugated and non-conjugated 6-beta-naltrexol are the major metabolites found in plasma, urine and feces. The half-life of elimination of parent naltrexone is 2.9 hours and 8.8 hours for 6-beta-naltrexol, which is a weak antagonist at the mu opiate receptor [27,29].

Naltrexone Depot consists of microspheres of poly (D, L-lactide) and naltrexone, administered by intra-muscular injection. The microspheres are mixed with a diluent containing water, mannitol, carboxymethylcellulose and polysorbate 80 to form a suspension for injection. Once injected, the suspension forms a solid gel pellet with the rate of release proportional to the surface area of the gel pellet, the loading of naltrexone in the microspheres and the porosity of the microspheres. The polylactide polymers are broken down to monomers by hydrolysis, releasing naltrexone and ultimately being metabolized to carbon dioxide and water.

### Statistical analysis

Concentration-time data were analyzed by noncompartmental techniques using WinNonlinProfessional 3.1. The pharmacokinetic parameters calculated were C_max_, t_max_, AUC_0-t_, AUC_0-last_, and the metabolite to parent AUC_0-t _ratio.

Drinking data were analyzed using the univariate procedure of SAS, version 8.2. Changes in liver function tests were evaluated using a one-way analysis of variance comparing baseline values to end of study values.

## Results

### Subjects

Of the 17 subjects screened for study participation, 16 subjects (94.9%) met the inclusion/exclusion criteria and received study medication. Of the 16 participating subjects, 14 (87.5%) completed the study through week 6. Of the 2 subjects (12.5%) who did not complete the study, one subject was lost to follow-up while the other was unwilling to complete the study due to the time required. The mean age of the participants in the study was 49 years with a range of 27–60 years. Thirteen (81%) of the subjects were male and 10 (62.5%) of the subjects were Caucasian. Demographic and baseline clinical characteristics are presented in Table 2.

**Table 2:** Baseline Demographic and Clinical Characteristics

**Characteristic**	
Age in yr, mean (SD)	49.2 (13.4)
Gender, N (%)	
Male	13 (81.3)
Female	3 (18.8)
Ethnicity, N (%)	
Caucasian	10 (62.5)
Black	5 (31.3)
Hispanic	11 (6.3)
DSM-IV Alcohol Dependence Criteria Met, mean (SD)	5.4 (0.7)
Baseline Heavy Drinking Days, mean (SD)	10.9 (3.5)
Baseline Drinks Per Drinking Day, mean (SD)	8.4 (3.5)
Baseline Heavy Drinking Days, mean (SD)	8.1 (3.8)

### Safety measures

As shown in Table 3, there was no significant difference between patients' vital signs and liver function tests at the beginning of the study and at the completion of the study.

**Table 3:** Vital Signs and Liver Function Tests

**Parameter**	**Pre-Treatment (= day -7) mean ± sd**	**Post-Treatment (last visit) mean ± sd**
Number of Patients	16	14
Systolic Blood Pressure, mm Hg	135 ± 13	132 ± 10
Diastolic Blood Pressure, mm Hg	85 ± 9	83 ± 6
Pulse, beats/min	80 ± 5	79 ± 9
AST, IU/L	31.9 ± 14.6	27.6 ± 22.7
Total Bilirubin, mg/dL	0.6 ± 0.3	0.6 ± 0.3

Over the course of the study all 16 subjects had 1 or more adverse events. A total of 15 subjects had injection site adverse reactions. Table 4 shows the injection site adverse events experienced by subjects in the study. Three subjects experienced drainage from their injection site. The drainage fluid of 2 of the subjects was available for culture and in both cases did not grow out any organisms. Of the 198 adverse events reported, 17 were rated severe: nausea, flatulence, gastrointestinal pain, fatigue, lethargy, somnolence (2 reports), depression, irritability, headache (4 reports from 3 subjects), back pain, injection site mass, injection site pain and elevated GGT. There were no serious averse events. As shown in Table 5, there were 13 reported changes in biochemical markers that were outside of normal ranges.

**Table 4:** Adverse Injection Site Events

Adverse Event*	**Mild**	**Moderate**	**Severe**
Bruising	2	0	0
Inflammation	1	1	0
Mass	3	3	1
Pain	3	6	1
Pigmentation Change	2	1	0
Site Reaction NOS	2	1	0

*All values, N (%)

**Table 5:** Laboratory Value Abnormalities

**Adverse Event**	**% Reporting Adverse Event**
Blood Lactate Dehydrogenase Increased	31.3
Gamma-Glutamyl Transferase Increased	18.8
Red Blood Cells Present In Urine	12.5
Proteinuria Present	12.5
Alanine Aminotransferase Increased	12.5
Blood Creatine Increased	12.5
Hemoglobin Decreased	12.5
Hematocrit Decreased	6.3
Lymphocyte Count Decreased	6.3
Neutrophil Count Increased	6.3
Protein Total Increased	6.3
Aspartate Aminotransferase Increased	6.3
Uric Acid Increased	6.3
Urine Analysis Abnormal NOS	6.3
White Blood Cell Count Increased	6.3
White Blood Cells In Urine	6.3

### Drinking measures

All measures of drinking declined from the baseline period to the 6-week treatment period: proportion of drinking days from 0.77 (s.d. 0.25) to 0.46 (0.33) (signed rank; S = -47.5, p = 0.0044), drinks per drinking day from 8.4 (s.d. 3.5) to 4.9 (s.d. 3.4) (S = -64, p = 0.0002) and proportion of heavy drinking days from 0.58 (s.d. 0.27) to 0.25 (s.d. 0.27) (S = -56, p = 0.0004).

### Pharmacokinetics

The pharmacokinetic results are based on weekly plasma sampling for six weeks on day 0, 7, 14, 21, 28, 35 and 42 following the single dose administration of 300 mg (2 × 150 mg) Naltrexone Depot. On Day 0 blood samples were drawn prior to dosing of Naltrexone Depot. Pre-dose concentrations reflect naltrexone and 6-beta-naltrexol plasma concentrations following multiple administration of 50 mg naltrexone oral tablets from study days -4 through -1, which were not expected to influence levels in subsequent samples. Pre-dose plasma concentrations on day 0 were set to O for the calculation of AUCs and were excluded from the reporting of t_max_. For naltrexone, mean (range) C_max _value was 2.640 (0.57–11.02) ng/mL. Values for t_max _were variable and ranged from 168 hr to 1,008 hr (median: 540 hr) post-dose. In terms of exposure, mean (range) values for AUC_0–28 _and AUC _0–42 _were 829 (149–3,332) ng/hr/mL and 1089 (341–3899) ng/hr /mL, respectively. For the major metabolite, 6-beta-naltrexol, the mean (range) C_max _value was 8.71 (0.99–41.13) ng/mL. Values for t_max _ranged from 168 hr to 1,008 hr (median: 336 hr). The mean 6-beta-naltrexol to naltrexone ratios were 3.5 for AUC_0–28 _and 3.4 for AUC_0–42_. The mean (range) values for AUC_0–28 _and AUC_0–42 _were 2528 (606–8872) ng/hr/mL and 3286 (988–8872) ng/hr/mL, respectively. Plasma concentrations of 6-beta-naltrexol were approximately 3-fold higher than naltrexone at all sampling times.

**Figure 3:**
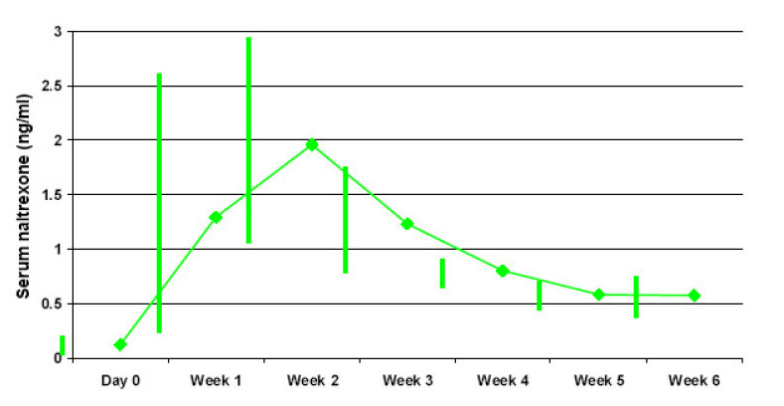
Serum Naltrexone Levels.

Fourteen (88%) subjects had detectable plasma concentrations of naltrexone at 42-days post-dose, 1 (6%) subject had a detectable concentration at their last visit 35-days post-dose and 1 (6%) subject had a detectable concentration at their last visit 14-days post-dose. The mean concentrations of naltrexone ranged from 0.58 ng/mL to 2.04 ng/mL at each sampling time after administration; see Figure 3

**Figure 4:**
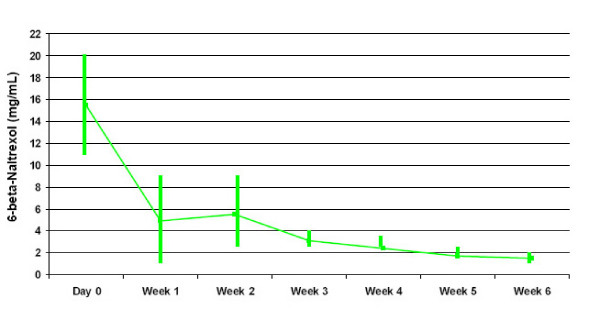
6-beta-Naltrexol Levels.

Thirteen (81%) subjects had detectable 6-beta-naltrexol concentrations at 42-days post-dose, 1 (6%) subject had a detectable concentration at their last visit 35-days post-dose, 1 (6%) subject had a detectable concentration at their last visit 14-days post-dose and 1 subject had a detectable concentration through 28 days post dose, after which 6-beta-naltrexol was undetectable. The mean concentrations of 6-beta-naltrexol ranged from 1.51 ng/mL to 5.52 ng/mL at each sampling time after administration; see Figure 4.

## Discussion

The results from this 6-week, single site, open-label study to investigate the safety and pharmacokinetics of Naltrexone Depot showed it to be safe, well tolerated and led to sustained plasma levels of naltrexone. The overall completion rate for study participants was 88%. The 2 subjects who did not complete the study were not lost because of adverse effects.

Injection site complications were by far the most common side effect experienced by subjects participating in this study. Of the side effects experienced the most severe were drainage from the injection site. In the absence of any evidence of infection and given the history of inflammatory reactions from naltrexone injections [30], these cases of drainage were determined to have been caused by a local inflammatory reaction. Although this rate of drainage was concerning, it is important to note that none of the subjects required hospitalization, the drainage was self limiting, and the subjects were remarkable unconcerned about it. Other common side effects were gastrointestinal, nervous system, and changes in metabolic markers, but these occurred at a similar rate as in other naltrexone trials in alcohol and heroin dependent subjects [5,11,31].

The mean plasma concentration across all sampling times ranged from 0.12 ng/mL to 2.04 ng/mL. On the last sampling day (day 42) all subjects available for testing had detectable plasma concentrations of naltrexone. These results are encouraging and show that the depot formulation of naltrexone can provide plasma concentrations of active drug for over 30 days. For a depot formulation of naltrexone to be accepted in clinical practice, a duration of action of at least 1 month is probably necessary [32]. This is the case because it limits the number of visits a patient has to make to their health care provider and limits the number of injections they must receive. Although detectability for the study duration is important, further information is needed on what constitutes an optimal level.

Plasma levels of 6-beta-naltrexol were 3 times higher than naltrexone levels at all sampling times. When naltrexone is administered through the IM route it bypasses 1^st ^pass metabolism, which leads to a lower ratio of 6-beta-naltrexol to naltrexone [33]. It is still unclear how much 6-beta-naltrexol contributes to the efficacy and adverse effects of Naltrexone Depot, but the lower ratio of 6-beta naltrexol to naltrexone may reduce adverse effects which are due to the 6-beta-naltrexol. In a preliminary study looking at the relationship between 6-beta naltrexol levels and the incidence of subjective side effects following oral administration of 50 mg of naltrexone, subjects who experienced more side effects had significantly higher urinary levels of 6-beta-naltrexol [33,34]. These side effects included headache, nausea, anxiety and spontaneous erection. Overall, subjects had a 10:1 urinary ratio of 6-beta-naltrexol to naltrexone. It has been suggested that 6-beta-naltrexol contributes to the continuing antagonism of heroin's effects due to the fact that its blood levels are consistently higher than naltrexone's after depot administration [19]. The pharmacological properties and activity of 6-beta-naltrexol need to be investigated further before a definitive answer can be given on the role of 6-beta-naltrexol in its ability to modulate the response to alcohol and heroin.

Subjects participating in this study experienced a decrease in drinking days, drinks per drinking day, and heavy drinking days. These results are consistent with the study by Kranzler [5](1998) using a different depot formulation of naltrexone. In that study there was a clinically significant effect on the frequency of heavy drinking days. The results from these 2 studies provide the foundation for large multi-site studies on the efficacy and safety of Naltrexone Depot.

Limitations of this study include the fact that this it was small, single site, open-label, and the sample is primarily older and male. The lack of a placebo group makes it difficult to assess the contribution of Naltrexone Depot to the reduction in drinking. Nonetheless, the results from this open-label study are encouraging and warrant further evaluation of Naltrexone Depot as a treatment option for alcohol dependent patients. Approaches such as this that optimize adherence have the potential to maximize the efficacy of naltrexone in the treatment of alcohol dependence.

## Competing interests

Funding for this study was provided to Gantt P. Galloway under a contract from DrugAbuse Sciences.

## Authors' contributions

GG carried out study procedures, analyzed data, and participated in design of the study. MK carried out study procedures. RC analyzed data. DS carried out study procedures. All authors read and approved the final manuscript.

**Figure 2:**
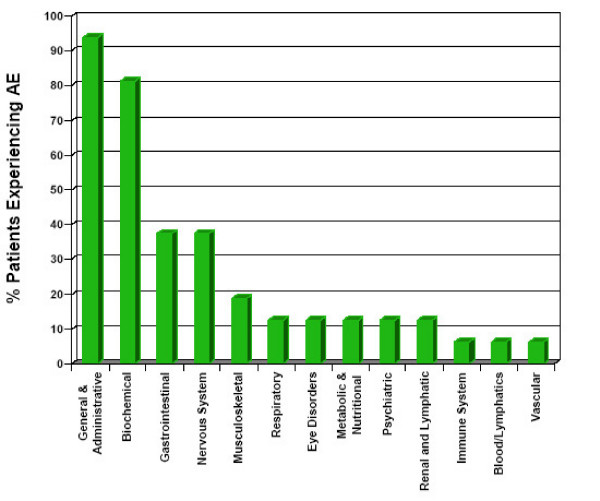
Adverse Events by Body System.

## Pre-publication history

The pre-publication history for this paper can be accessed here:


